# A Feasibility Study of In-Situ Damage Visualization in Basalt-Fiber Reinforced Polymers with Open-Source Digital Volume Correlation

**DOI:** 10.3390/ma16020523

**Published:** 2023-01-05

**Authors:** Frank Fischer, David Plappert, Georg Ganzenmüller, Ralph Langkemper, Victoria Heusinger-Hess, Stefan Hiermaier

**Affiliations:** 1 Department of Sustainable Systems Engineering (INATECH), Albert-Ludwigs-Universität, 79110 Freiburg, Germany; 2 Fraunhofer Institute for High-Speed Dynamics, Ernst-Mach-Institut (EMI), 79104 Freiburg, Germany

**Keywords:** digital volume correlation, µCT, BFRP, in-situ

## Abstract

This work analyses damage formation within the bulk of basalt fiber-reinforced polymers (BFRP) by means of open-source Digital Volume Correlation (DVC). Volumetric image data were obtained from conventional in-situ X-Ray computed micro-tomography (µCT) of samples loaded in tension. The open-source image registration toolkit Elastix was employed to obtain full 3D displacement fields from the image data. We assessed the accuracy of the DVC results using the method of manufactured solution and showed that the approach followed here can detect deformation with a magnitude in the order of a fiber diameter which in the present case is 17 µm. The beneficial influence of regularization on DVC results is presented on the manufactured solution as well as on real in-situ tensile testing CT data of a BFRP sample. Results of the correlation showed that conventional µCT equipment in combination with DVC can be used to detect defects which could previously only be visualized using synchrotron facilities or destructive methods.

## 1. Introduction

Conventional analysis of mechanical loading experiments is based on surface observation of the sample during the test using, e.g., Digital Image Correlation (DIC) [1] or post-mortem analysis of the failed sample using micro-graphs. The former does not allow observation of deformation in the interior of the sample during the test, while the latter analyses the sample in an unloaded state. Both approaches are therefore not entirely representative of the conditions within the bulk. 

In-situ µCT experiments overcome these limitations by performing mechanical loading experiments within a CT-scanner to acquire 3D images of a sample under load. The result of this procedure is a sequence of 3D images, which differ in their internal deformation field relative to an initial, unloaded reference state. The displacement field that describes the deformation map between the two states may be obtained using Digital Volume Correlation (DVC) and allows the determination of full 3D displacement fields between a set of volumetric images. The method was first presented by Bay et al. [2] to estimate strain occurring during compression of trabecular bone. More recently, it has been used on a routine basis in biological and biomedical research [3,4], as well as in geology [5,6]. In engineering, DVC is increasingly often applied on polymers and aluminum foams [7,8] as well as metal-alloys [9,10]. Interesting targets to apply DVC to are fiber-reinforced polymers (FRPs) due to the complexity of the stress states and failure mechanisms in these heterogeneous and anisotropic materials. However, these FRPs are challenging to investigate because of the small fiber diameters and the typically poor image contrast, which is a result of the low X-ray attenuation difference between fibers and matrix. While high resolution µCT setups are able to resolve individual fibers, it is still a challenge to acquire high quality images with fiber diameters below 20 µm in low-contrast FRPs [11,12]. Therefore, we chose a basalt fiber-reinforced polymer (BFRP) for this study. Its chemical composition includes heavy oxides (e.g., 11–12% Fe2O3 [13]), which increase the difference in attenuation between fiber and matrix and therefore improve contrast. This makes BFRP a suitable candidate for in-situ testing in combination with DVC as these intensity variations that create image contrast act as naturally occurring 3D speckle pattern.

Several commercial DVC algorithms are available. Products from Volume Graphics [14], Thermo Fisher [15] and LaVision [16] offer high quality, state-of-the art DVC algorithms. As these tools are proprietary, without released source codes, they are not ideally suited for academic research. A number of open source alternatives are available, e.g., TomoWarp2 [17] or FIDVC [18]. However, these mainly address correlation problems specific to a certain application. Therefore, they are often not generally applicable to a wide variety of correlation problems and, owing to their academic background, somewhat limited in computational performance. An exception to this general observation is the open source software Elastix [19,20] which is in large parts based on the Insight Segmentation and Registration Toolkit (ITK) [21]. We emphasize that Elastix implements DVC algorithms, and is not only a registration tool which aligns image edges, an erroneous opinion we have often encountered. For a review concerning the similarity of image correlation and registration algorithms, see [22]. Elastix originates from a medical background and has been applied and validated for medical applications. It offers many user-adjustable parameters to control the image correlation process. While this makes it a flexible tool, parameter tuning according to image contrast, feature size and the expected magnitude of deformation are essential to receive the best correlation and reliability of the analysis of in-situ tensile tests of engineering materials. Addressing this by means of correlation of a manufactured solution was the first goal of this work. A second goal was to show that damage localization in FRPs can be derived directly from evaluation of a strain tensor computed from the DVC displacement field. 

A number of in-situ experiments on FRPs have already been done, mostly using synchrotron X-ray sources [11] due to their ability to produce highly collimated, monochromatic and high brilliance X-ray beams [23,24,25]. These studies were able to capture small defects on the length-scale of one fiber diameter of approximately 10–20 μm. However, identification of defects was achieved by direct visual inspection. Here, we intended to utilize DVC to automate the process of defect detection by using a suitable physically based strain metric. In addition, we used conventional laboratory µCT systems, as these are more readily available than access to synchrotron beam-time.

Our work focused on how a tensile deformation field, derived from DVC of conventional CT images, assists in the detection of local damage in basalt fiber reinforced polymers.

The remainder of this article is organized as follows. We begin with presenting the applied methodology, starting with a description of the FRP samples and their mechanical properties. Following this, we describe our in-situ X-Ray CT setup, introduce the DVC algorithm implemented in the Elastix toolkit and establish an accuracy estimation based on the analysis of a synthetic deformation applied to real CT data. Finally, we describe DVC performed on CT images of a BFRP sample after tensile testing to show that damage features with a minimum size of one fiber diameter can be both identified and tracked from strain maps throughout the full sample volume. 

## 2. Materials and Methods

### 2.1. Test Specimens

**Figure 1:**
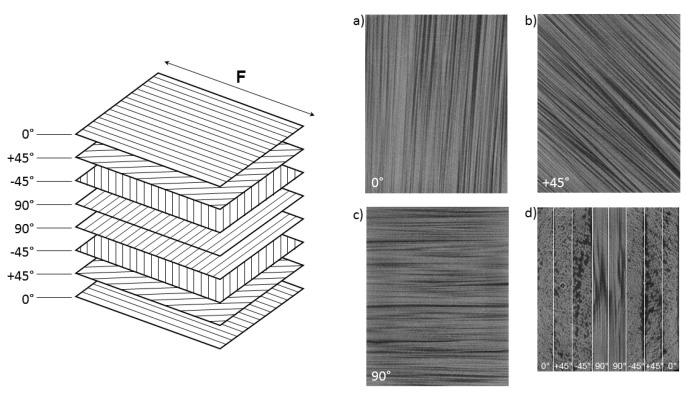
Sketch of the specimen layout to form the quasi-isotropic laminate with CT images of the respective layers. Naming of the fiber orientation is with respect to the direction of the applied tensile force (F). Major orientations are 0° (**a**), ±45° (**b**) and 90° (**c**). In an axial CT image (**d**) all eight individual plies are visible.

Laminated composite sheets were provided by INCOTELOGY GmbH, Pulheim, Germany. The composite was made from prepregs of basalt fibers with a filament diameter of 17 μm and a low-viscosity epoxy resin system. The Youngs’ modulus of the bare fibers according to ASTM-D2101 [26] was 92 GPa as reported by the manufacturer. The epoxy resin system used was the CP003 (prepreg identifier: CM-Preg T-B- 300/280 CP0031 32) by c-m-p GmbH 52525 Heinsberg, Germany. The composite was cured in an autoclave process with a 30 min ramp up time from ambient conditions to 120 °C at a pressure of 3 bar, followed by a 120 min plateau and a 30 min ramp down to ambient conditions. The resulting fiber volume density was 60%. The laminate was made from eight individual plies, each being strongly anisotropic due to their unidirectional fiber orientation. The orientation of these plies in the laminate was (0/±45/90s; see Figure 1) which resulted in a quasi-isotropic material behavior on a macroscopic level. The total laminate thickness was 1.6 mm. Specimens were cut from the sheet to a cross sectional area of 3 mm^2^ and a gauge length of 5 mm using a diamond coated saw blade. 

**Figure 2:**
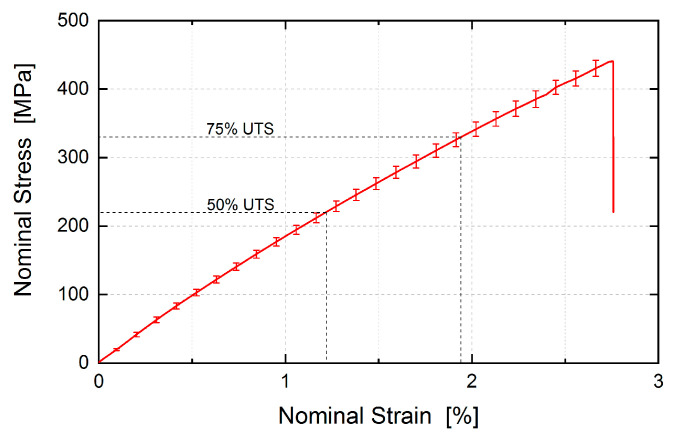
Stress-strain behavior of the quai-isotropic BFRP laminate. The vertical error bars represent the standard deviation of the individual tests over the test series [26]. The UTS is 441±33 MPa. CT scans for subsequent examination with DVC were performed at two different loading states corresponding to 50%, and 75% UTS.

Figure 2 shows the tensile behavior of the quasi-isotropic BFRP in a tensile test according to ASTM D 3039 standards with an ultimate tensile strength (UTS) of 441 ± 33 MPa. For further details on material properties, see the in-depth characterization of this material [27].

### 2.2. In-Situ CT Imaging under Tensile Load

**Figure 3:**
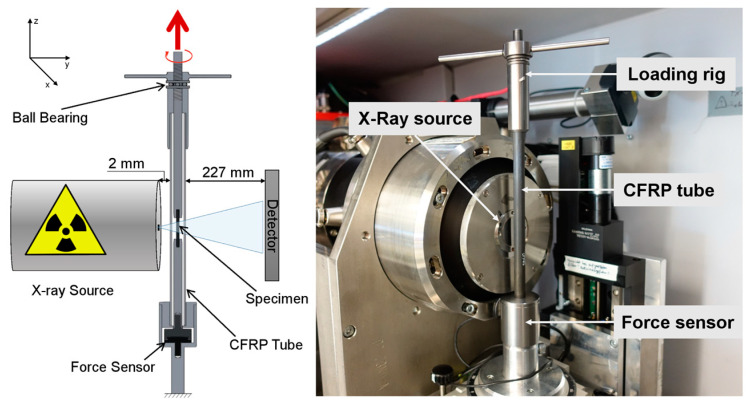
Sketch (**left**) and picture (**right**) of the in-situ CT experiment setup. By turning the wing nut at the top of the loading rig, the inner rod is pulled upwards and causes tensile stress in the specimen. To maximize magnification, the distance between X-Ray source and loading rig wall is at a minimum of 2 mm (sketch not to scale).

A custom test rig was constructed to perform tensile loading of a miniaturized specimen inside the limited space of the measurement chamber of the CT. As shown in Figure 3, the specimen was loaded by turning a threaded nut attached to the upper part of the specimen. The lower part of the specimen was connected to a base comprising a force sensor, which continuously records the loading state of the specimen during CT image acquisition. The base was connected to the threaded rod by a hollow tube made of carbon fiber reinforced polymer and an axial ball bearing, to provide a path for the reaction forces. This setup allowed loading the sample in tension without torque.

CT-scans were performed using a CT350 setup from MacroScience Technology GmbH (München, Germany), equipped with a L10711 micro-focus X-Ray tube and the CMOS Flat Panel Sensor C7942SK-05 (12-bit, 2304 × 2304 px, 50 μm pixel pitch) both manufactured by Hamamatsu Photonics K.K. (Hamamatsu City, Japan). The X-ray source was operated at 80 kV/80 μA. Geometrical magnification in cone beam geometry by a factor of 32.4 with a 2 × 2 binning led to an effective voxel size of 3.2 μm^3^. In this way, a total of 1200 projections were recorded over the course of 4 h.

The in-situ investigation consisted of two scans of the specimen in a sequence of increasing tension stress levels, see Figure 2. At first, the sample was in a nearly unloaded condition. To ensure alignment with the vertical axis, a preload of 5% UTS (66 N) was applied. Subsequently, the sample was loaded and scanned at 50% UTS (660 N) and finally 75% UTS (990 N).

### 2.3. Digital Volume Correlation

DVC computes the displacement field that describes the deformation of a solid sample using volumetric images such as CT images. It was first described by Bay et al. to compute strain maps in compressed bone [2]. In contrast to DIC, DVC does not rely on an artificial pattern to track displacements, but instead must make use of local intensity variations arising due to the changes in X-ray absorption caused by material composition and microstructure of the sample under investigation.

Following the conventions adopted by Hild et al. [28], the Elastix algorithm finds the displacement field u which links a reference image f containing grey scale intensities to a deformed image g. Denoting the spatial position as *x*, a conservation statement about the grey scale intensities may be expressed as:

(1) f(x)=g[x+u(x)]

Assuming that, within a small zone of interest (ZOI), *u* is locally constant, the solution for *u* can be found by maximizing the cross correlation, hence the name volume correlation.

(2) $$\left(f⋆g\right)\left(u\right)=\int_{ZOI}f\left(x\right)g\left(x+u\right)\;dx$$

The correlation process is numerically solved in an iterative manner. The transformation is defined by a uniform grid of control points with the spacing between the grid nodes defining the grid density and the degrees-of-freedom the transformation possesses. In Elastix, the grid spacing should be adapted hierarchically in most cases. In the early stage, a coarse grid is used to identify large deformations. This guides the solution process to achieve convergence to the global minimum. Subsequently, the grid resolution is refined. The spacing at the finest resolution level is termed Final Grid Spacing and must be larger than one pixel to provide an intensity distribution such that u is determined unambiguously. To recover the full resolution, interpolation between the grid points is used to obtain an approximate *u*(*x*) at every voxel position. 

The actual algorithms implemented in Elastix are far more complex than the here presented summary, involving a large number of parameters. An exhaustive description is beyond the scope of this paper and can be found elsewhere [29].

### 2.4. DVC of a Manufactured Solution—Proof of Concept

The accuracy of the DVC solution is difficult to quantify in real experiments. Residuals, i.e., the deviation field between the reference and back-displaced deformed image can only be used to monitor convergence of the algorithm on an optimum, which potentially is a local minimum but not the global true solution. Therefore, we instead considered the method of manufactured solutions [30], i.e., we generated a deformed image by prescribing a three-dimensional, local deformation on a sub-volume of the unloaded reference CT image. The prescribed deformation field $\tilde{u}$ is given by a smooth polynomial with compact support.

$${\tilde{u}}_{x}=0$$

$${\tilde{u}}_{y}=0$$

(3) $$\begin{array}{c} {\tilde{u}}_{z}\left(r,\Delta z\right)\\ =\{\begin{array}{c} \frac{\Delta z}{\left|\Delta z\right|}\frac{r\;{\left(r^{2}-h^{2}\right)}^{4}}{h^{4}}\;\;\;\;\;if\;r<h\\ \;\;0\;\;\;\;\;\;otherwise \end{array} \end{array}$$

Here, $\Delta x=x-x_{0}$, $\Delta y=y-y_{0}$, and $\Delta z=z-z_{0}$ are distances of a location in the image with respect to the centre of the deformation at $\left(x_{0},y_{0},z_{0}\right)$, for which we chose the centre of the volumetric image. The magnitude of this distance is $r=\sqrt{\Delta x^{2}+\Delta y^{2}+\Delta z^{2}}$. The size of the deformed domain is a sphere with radius h=12 px corresponding to one fiber diameter. Note that we only introduced displacements in the z-direction, mimicking a tensile experiment. The resulting strain, $\epsilon_{z}=\frac{du_{z}}{dz}$, attains a value of one at the center of the deformation while the displacement amplitude ranges from −3.5 to +3.5 px, ensuring overall volume preservation due to the deformation field symmetry. 

**Figure 4:**
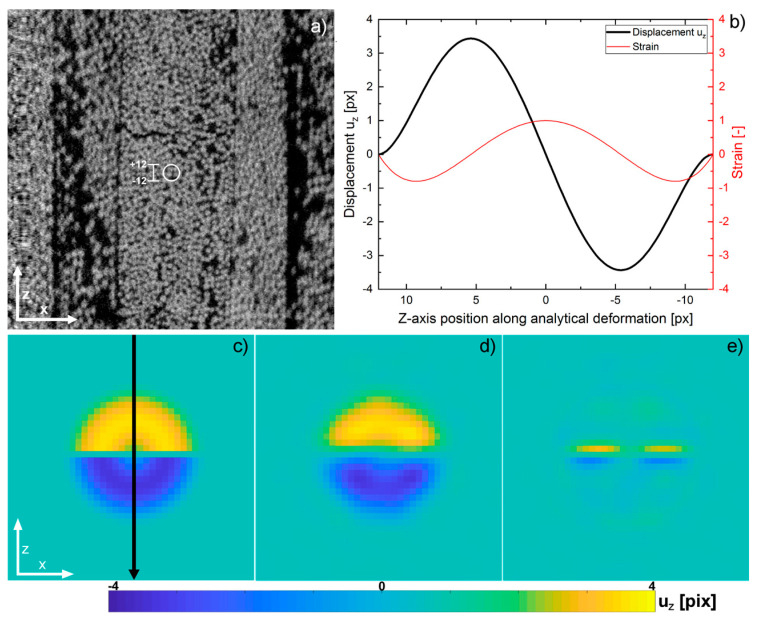
The region of the analytical deformation is marked in the reference CT slice (**a**). The displacement within this deformation ranges from −3.5 to +3.5 px with a max. strain of one in the center. The graph (**b**) shows a line plot through the center of the analytical deformation. The central xz-slice of the manufactured solution together with the position of the line profile (**c**), the displacement field obtained from DVC (**d**) and the residuals between the former (**e**).

Figure 4a shows the central slice of the reference CT image where the white circle depicts the area of the prescribed deformation in this particular slice, whereas Figure 4b contains a line profile representation of the prescribed deformation itself. The manufactured solution is applied to the initial reference image to yield the deformed image. A Gaussian noise distribution is added to the grey scale intensities of the deformed image to create varying image noise.

The displacement field, u, was obtained by DVC and subsequently compared to the manufactured solution. Improvement of the quality of the DVC process could be achieved by adjusting the set of parameters that define the correlation process as well as penalizing displacements that result from image artifacts and have no physical relevance. Figure 4d contains the displacement field as computed by the correlation with an optimized parameter set (for the optimization approach, see Appendix A). Both shape and amplitude closely resembled the manufactured solution. The residual image in Figure 4e however indicates limitations in the correlation if a large displacement gradient is present, such as close to the center of the manufactured solution, where displacement values change sign within a single pixel row. This uncertainty is bothersome, as both large gradients and maxima in the displacement field need to be preserved as accurately as possible, while reducing overshooting and subsequent ringing effects in vicinity to the deformation. The presence of such numerical artifacts could otherwise lead to false interpretation. A regularization term, where the influence of regularization is expressed as a weighted fraction relative to the similarity metric, suppresses these artifacts. The penalty term used is proportional to the Laplacian of the displacement field. This is referred to as bending energy penalty in Elastix [31,32] and it is defined as:

(4) $$P_{BE}=\frac{1}{P}\overset{}{\underset{{\tilde{x}}_{i}}{\sum }}\|\frac{\partial^{2}T}{\partial x\partial x^{T}}\left({\tilde{x}}_{i}\right){\|}_{F}^{2}\;=\frac{1}{P}\overset{}{\underset{{\tilde{x}}_{i}}{\sum }}\overset{3}{\underset{k,l,m=1}{\sum }}{\left(\frac{\partial^{2}T_{k}}{\partial x_{l}\partial x_{m}}\left({\tilde{x}}_{i}\right)\right)}^{2}$$

Here, T is the transformation and P is the number of points ${\tilde{x}}_{i}$. The tilde denotes the difference between a variable and a point over which a term is evaluated. The use of such a regularization agrees to some extent with the kinematic behavior of a physical material, where strong strain gradients do not occur naturally, as it requires energy to create them.

**Figure 5:**
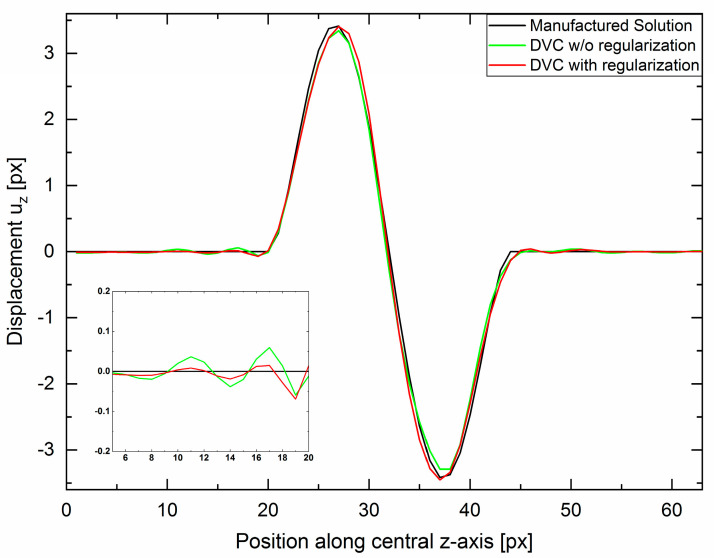
Line profile of the displacement from the central slice along the z-direction as indicated by the arrow in Figure 4c. Improvement of overshooting and ringing by use of regularization. The insert magnifies the left tail indicating a reduction in overshoot and subsequent ringing. No decrease in maximum amplitudes can be identified by using regularization.

The approach implemented in Elastix is a multi-resolution, multi-metric scheme with the regularization acting as second optimizable metric next to the cross-correlation. The weighting of the regularization is adjustable at every resolution step. For the DVC leading to the results in Figure 4, a correlation with four levels of resolution was chosen. Weighting of the regularization was kept at 10% for the first three resolution cycles and increased to 15% for the last cycle. This led to no suppression of large deformation in early stages, and no loss of maximum amplitude of the deformation. During later stages of the correlation process, at original image resolution, local curvature is more heavily penalized. Figure 5 shows a line plot of the displacement field along the *z*-axis through the center of the deformation (corresponding to the arrow in Figure 4c. Regularization does not degrade peak magnitude but serves to reduce the oscillations at the tails. DVC without regularization shows ringing in the vicinity to the deformation, c.f. the inset of Figure 5. Regularization results in a second beneficial effect: as already mentioned, the exact displacement field $u_{z}$ changes sign when passing through the centre of the xz-plane and $u_{z}=0$ in the entire central xy-plane. However, without regularization, the mean displacement as recovered by Elastix in the central xy-plane is 0.30 px. By employing the regularization, this value is reduced by 50% to 0.15 px, therefore making the displacement field more reproducible even if strong gradients are present.

## 3. Results

### DVC Application to In-Situ Tension Testing on BFRP Specimen

This section reports the DVC results obtained with Elastix using an optimized parameter set with respect to the correlation problem at hand (Identification of optimized parameter set, see Appendix A). The most severe change from default parameters was an increased control point spacing as a direct consequence of larger deformations that are expected when damage starts to form. Furthermore, regularization was introduced as it not only serves to reduce unrealistically large deformations in late stages of the correlation but also helps in diminishing parasitic displacements caused by the influence of artifacts and noise as will be shown in this section.

The physical metric that we chose derives from the deformation gradient $F=\frac{\partial u}{\partial x}+I$, which was computed using finite central differences of the displacement field with respect to the coordinate frame of the reference image. To remove any rigid rotation information, which is physically insignificant, the Green-Lagrange strain was then computed as $E=\frac{1}{2}\left(F^{T}F-I\right)$. Its isotropic part,${\;E}_{\mathrm{iso}}=\frac{1}{3}\left(E_{\mathrm{xx}}+E_{\mathrm{zz}}+E_{\mathrm{zz}}\right)\;I$, encodes volumetric strain information, while the deviatoric part, $E_{\mathrm{dev}}=E-E_{\mathrm{iso}}$, embodies information about non-isotropic strain states. Its magnitude, $E_{\mathrm{eq}}=\|E_{\mathrm{dev}}\|$, describes defects separating two half-spaces, such as a crack surface. This strain measure provides a simple scalar quantity to measure localized deformations. It is thus indicative of damage, but only in a qualitative way as we cannot accurately define a characteristic threshold, which distinguishes cracks from isolated regions with high strains. However, given that fiber-reinforced material failed on a macroscopic scale at strains of approximately 3%, it appears safe to conclude that if the strain measure $E_{\mathrm{eq}}$ exceeds twice this value, local damage is present. Similar other scalar metrics derived from the strain tensor could be employed to indicate strong local deformations, e.g., the largest eigenvalue. We chose $E_{\mathrm{eq}}$, because it is useful to indicate conditions where one eigenvalue of the strain tensor is very different from the other two eigenvalues, such as near an internal surface corresponding to a microcrack.

We note that the current established state of the art for localized damage is based on the residual of the DVC algorithm, i.e., a measure of the convergence. If convergence cannot be achieved, the residual is high, and it is assumed that this is due to the presence of a crack, which violates the inherent DVC assumption of a smooth displacement field. In our opinion, a strain measure, being a physical quantity, should be used to indicate cracks, instead of a non-convergence criterion of the algorithm.

**Figure 6:**
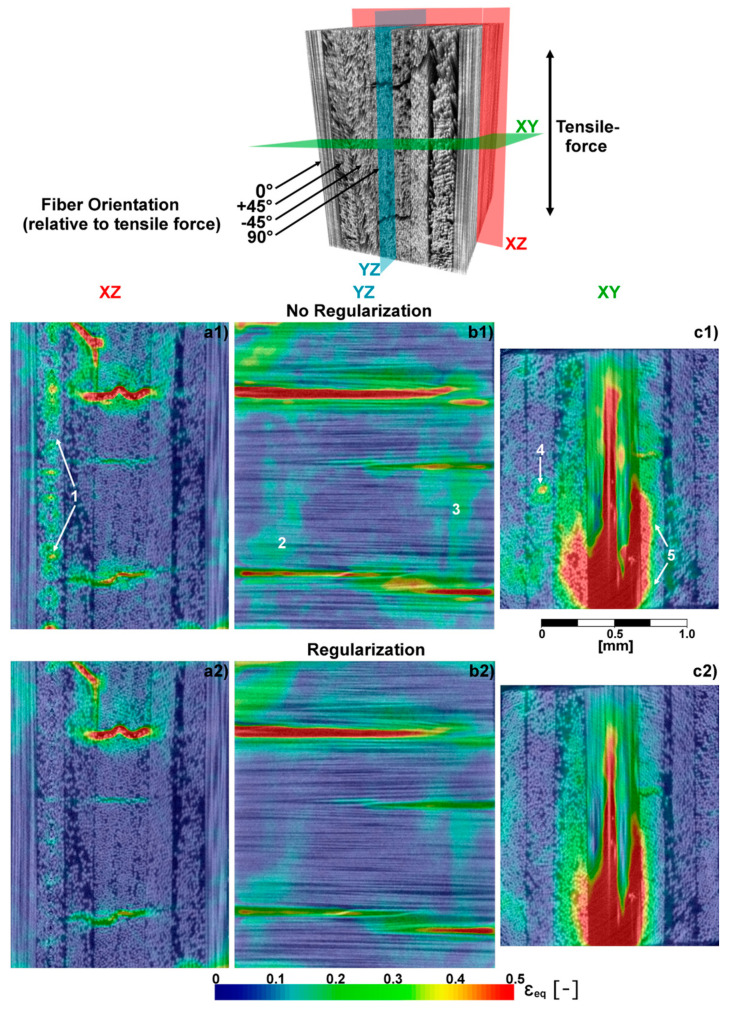
Top: Volume rendering with fiber orientation and force indicator. The coordinate system is adopted from Figure 4. Colored planes represent the positions of the 2D images shown in (**a1**–**c2**); (**a1**–**c2**) contain von-Mises equivalent strain quantities superimposed on the respective CT-slices. Landmarks (1–5) in (**a1**–**c1**) emphasize locations of ambivalent and erroneous strain quantities distinctly reduced by the implementation of the regularization (see (**a2**–**c2**)).

Figure 6 shows the equivalent strain obtained for the load case of 75% UTS. Here, the material gives the macroscopic impression of still being physically intact, but audible noises originating from failure within the material can be noticed. This means that the material has accumulated damage on the inside, but its strong 0°-plies on the outside are still withstanding the applied load. As expected, damage predominantly localizes in the central plies of the quasi-isotropic laminate. These plies are oriented in the 90° direction, i.e., the specimen is pulled apart perpendicular to the fibers of these plies. The observation of emerging damage in these plies agrees with the macroscopic characterization of this material [27], as both strength and failure strain are smallest for this direction compared to other fiber directions. Figure 6(c1,c2) show that cracks propagate into the ±45°-plies, but do not penetrate the outer 0°-plies.

To demonstrate the importance and capability of regularization to produce meaningful results in the presence of CT artifacts and evolving damage such as cracks, Figure 6 compares DVC results obtained with and without the use of regularization. The influence of ring artifacts was significantly reduced by introducing regularization. In the xz-plane, these artifacts manifest as vertical streaks of pseudo-strains (landmark 1), and are visible as circular structures in the xy-plane (landmark 4). In addition, large patches containing homogeneous strain values, c.f. landmarks 2 and 3, are reduced. Although these patches apparently correlate with areas of densely packed fibers, creating low contrast image regions and therefore ambiguous DVC results, it is not yet clarified whether these strain values also contain physical information to some extent. However, we can state that the chosen level of regularization removes ambiguous strain values, whilst preserving significant strain magnitude in damaged regions. Furthermore, regularization appears to reduce spill over to adjacent plies next to expanding inter-fiber cracks (landmark 5).

## 4. Conclusions

This work presents in-situ CT tensile tests on a specific composite polymer containing basalt fibers in combination with DVC. It was shown that current lab CT permits the acquisition of images with sufficient contrast and resolution to resolve single fibers, allowing in turn to successfully perform DVC. To this end, the open-source code Elastix was employed. A validation study was conducted by analysing at synthetic deformation field imposed on an actual µCT data set. This study showed that the DVC algorithm implemented in Elastix can indeed resolve a strongly localized deformation feature equivalent to a real displacement field under conditions close to failure for this class of material.

We found that it was important to guide the correlation process using a regularization term which penalizes strong gradients in the resulting displacement field. This improved correlation results in high gradient regions while conserving maximum amplitude displacement peaks.

Using real CT data sets acquired at 50% and 75% UTS, it was shown that CT-artifacts reduce the accuracy of the DVC displacement field. However, regularization helps to reduce the errors caused by these artifacts, resulting in high quality DVC results throughout the full 3D sample volume.

These results provide insights into the failure behavior of the materials in a way that is not possible from exterior surface observations only; for example, damage that starts as line features on the sample exterior form crack surfaces within the volume of the sample. The three-dimensionality of the CT data also makes it possible to view and track any damage features throughout the sample volume that do not extent to the sample surface. 

The main result of this work is that data from conventional µCT systems can be used for performing DVC analysis on fiber-reinforced materials, at least when applied to high-contrast composites such as basalt-fibers. Although synchrotron CT still represents the best method norm for obtaining CT images for DVC, it is no longer mandatory to perform DVC. In our opinion, the quality of the DVC displacement field should be high enough to perform an analysis of free surfaces created within the body, yielding quantitative data for calibrating continuum damage models of LeMâitre-type [33]. This, however, remains a topic for future studies.

## Figures and Tables

**Figure A1:**
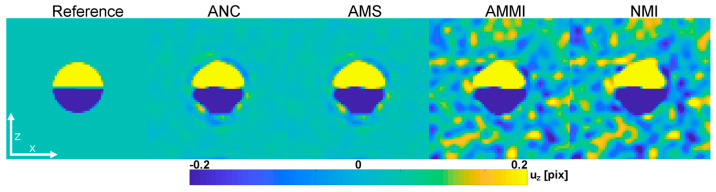
Representation of the reference displacement field (manufactured solution) and the displacement field as recovered by Elastix, with various definitions of the similarity metric. Reference illustrates the exact displacement field, i.e., the manufactured solution, while ANC, AMS, AMMI and NMI correspond to different metrics used in the DVC code Elastix.

**Figure A2:**
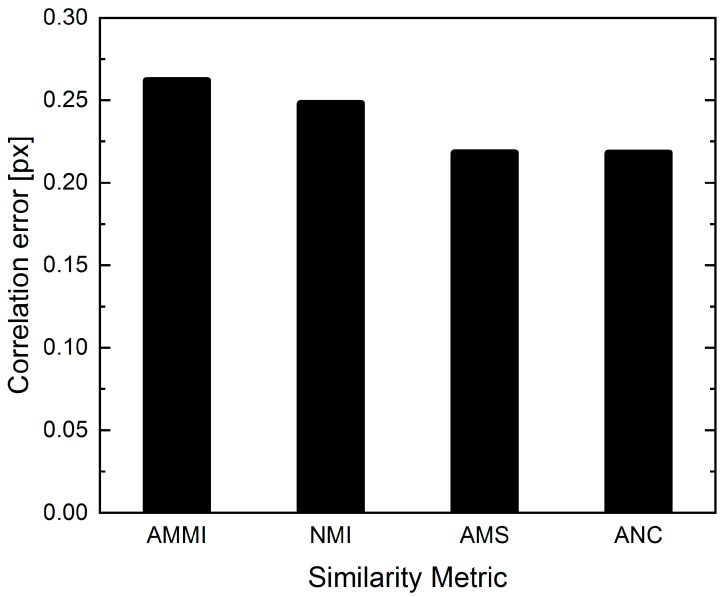
Correlation errors after DVC run with different similarity metrics.

**Figure A3:**
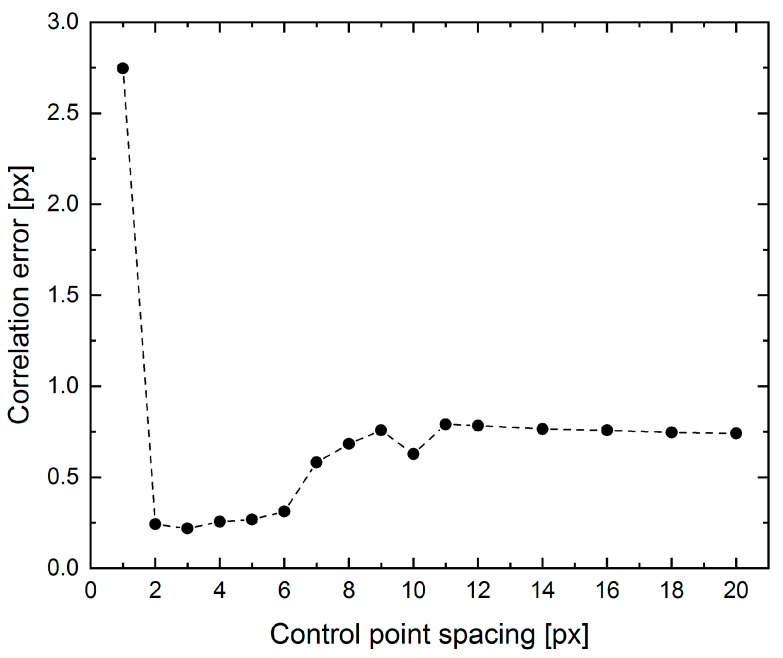
Effect of the control point spacing in the final resolution of the multiresolution approach, namely the final grid spacing, on the correlation error.

**Figure A4:**
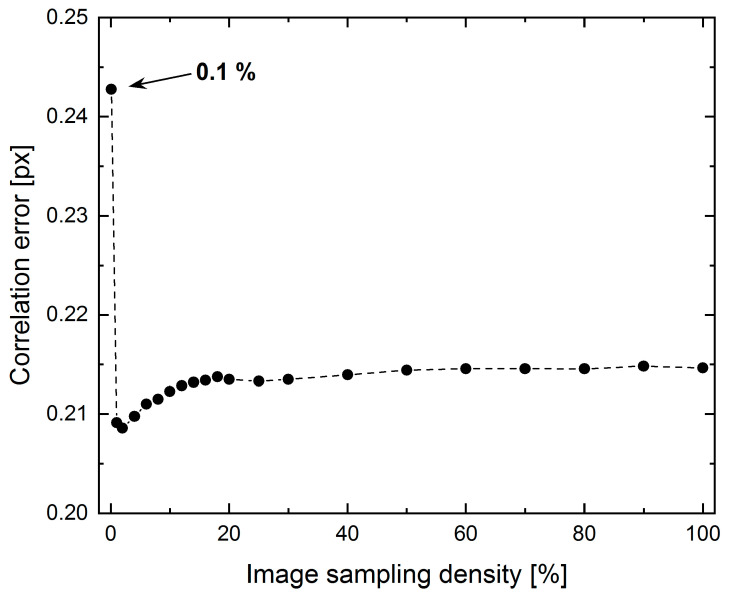
Correlation error with respect to the amount of image voxels used for similarity metric calculation after each iteration.

**Figure A5:**
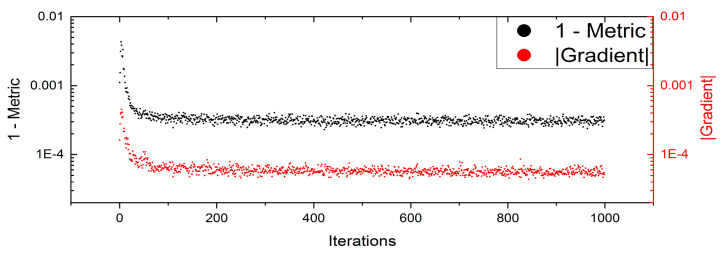
Exemplary evolution of the similarity metric and its gradient in the final resolution of a DVC run as a function of the number of iterations used.

## Data Availability

Not applicable.

## Appendix A. Elastix Parametrization Optimization for a Manufactured Solution

The displacement field u, which links reference and the artificially deformed image, was obtained by DVC and subsequently compared to the manufactured solution, yielding the error of the correlation result. This error is defined as the mean absolute error:

(A1) $$\mathrm{err}=\frac{1}{N}\overset{N}{\underset{i=1}{\sum }}\|u\left(x_{i}\right)-\tilde{u}\left(x_{i}\right)\|.$$

The mean absolute error is suitable for this problem, as we expected no significant outliers due to the smooth prescribed displacement field. The summation includes all discrete voxels which lie within the sphere of deformation (see Equation (3)). In addition, three pixels surrounding the sphere were added to incorporate errors resulting from overshooting in close proximity of the displacement, to the error calculation. Thus, the error is the average magnitude of the difference between prescribed and DVC-computed displacement fields in units of pixels. By adapting the DVC parameters, the correlation error can be minimized. In the following, we introduce these parameters.

### Appendix A.1. Metric

Metrics represent similarity measures in Elastix that determine the quality of alignment at the current stage of the correlation. The value of the metric is minimized during the course of the correlation in an iterative fashion. Mutual Information (Advanced Mattes Mutual Information, AMS) is a general similarity measure used for the registration of both mono- and multi-modal images [34,35]. The normalized version (Normalized Mutual Information, NMI) was included in this study as it was previously shown to improve image alignment for multi-modality images and increased robustness [36] compared to its non-normalized counterpart. The Mean Squared Distance (Advanced Mean Squares, AMS) is a similarity measure for mono-modal image registration and is calculated from the mean squared intensity differences between reference and deformed image. In contrast to Mutual Information, AMS is only suitable for mono-modal images, since the same intensity distribution is expected for both volumes. The Normalized Correlation Coefficient (Advanced Normalized Correlation) is well known in image registration [37]. It assumes a linear relation between the image intensities and is therefore well suited for mono- and intra-modal CT-images. This similarity measure is also commonly found in both commercial [38] and open-source [39] correlation algorithms. ANC computes the pixel-wise cross-correlation, normalized by the square-root of the auto-correlation of both the reference and the deformed image.

As all our images are CT-images had an equal dynamic range of intensities, all four similarity metrics were valid to use here. Both Figure A1 and Figure A2 indicate that AMS and ANC fare better than the other similarity measures, AMMI and NMI. The latter two result in strong oscillations outside of the region which contains the displacement, i.e., caused solely by image noise. Thus, AMS and ANC are deemed more robust. Although they give similar results, we settle for using ANC for the remainder of this study as it is also commonly employed in other DVC codes [38,39].

### Appendix A.2. Control Point Spacing

We chose cubic B-splines as the basic functions to describe the deformation field. As B-splines are locally supported, the number of control points, or knots, directly affects the degrees-of-freedom, of the deformation field. Therefore, the control-point spacing density in the 3D image is a major factor that controls the quality of the DVC solution. To capture a wide range of displacement magnitudes and increase the robustness of the registration process, a multi-resolution approach was employed [40]. The images were subjected to a fourfold hierarchical sequence of Gaussian smoothing operations. Initially, the strong smoothing, corresponding to a large Gaussian kernel bandwidth was employed. This ensured that only long-wavelength deformations with large spatial extent were recognized, such that a good initial guess for the overall deformation field was established. This made the correlation less prone to falling into a local minimum. Iteratively, the kernel bandwidth was reduced down to the original resolution of the images. The displacement field was represented using B-splines, which were defined on a grid of control points. The grid density, i.e., its spatial resolution, was also successively increased as the iterative algorithm for finding the displacement field converges. Starting with a coarse grid increased the robustness with respect to finding large deformations, whereas fine-scale deformations were captured in later stages at finer grid spacing. As the default values for the refinement scheme of the grid spacing were used, the parameter under review was the control point spacing in the final resolution of the multiresolution approach, namely the final grid spacing.

We found that the final resolution of the B-spline control grid spacing strongly affected the quality of the displacement field solution. A clear optimum could be found which can be explained as follows: a coarse final grid implies a low uncertainty at the cost of resolution and possible negligence of small displacements while a fine grid will produce high resolution results with higher degrees of uncertainties due to fewer voxels being considered, a relative increase in the influence of image noise and artifacts and therefore possibly allowing the transformation to have too much freedom. The influence of the user-adjustable parameter Final Grid Spacing (FGS) is displayed in Figure A3. An optimum was reached for an FGS of three voxels, corresponding to one fourth of the spatial extent of a fiber diameter. Even finer grids resulted in higher errors due to insufficient statistics, while coarser grids failed to accurately recover small, strongly localized displacements.

### Appendix A.3. Image Sampling Density

To solve for the displacement field during the iterative correlation, the metric would ideally include all voxels of the image. However, high-resolution CT images can easily exceed 10^9^ voxels, making the computation quite time consuming. To address this issue, Elastix implements a stochastic approach calculating the metric from a small subset of spatial samples with the locations randomly chosen at each iteration. It has been shown that this approach of Adaptive Stochastic Gradient Descent (ASGD) [41,42] yields both convenient convergence properties and fast computation times. In particular, the stochastic sampling nature (random re-sampling) can make the similarity metric a smoother function of the transform parameters which is beneficial for both accuracy and robustness of the optimization process [43,44].

The sampling rate per iteration not only affects computation time, but rather influences the convergence properties of the optimization, as indicated in Figure A4, and therefore the quality of the result for a fixed number of iterations. It is shown that a sampling density of only just more than 0.1% of all image pixels sufficed to achieve good results. However, such a small value is near the steep rise of the error towards smaller values and results are therefore not trustworthy. As the error does not increase significantly when increasing the sampling density, we use a value of 10%. This compromise provides a significant increase in computation efficiency whilst also appearing safe in terms of a robust and reproducible result.

### Appendix A.4. Correlation Convergence Threshold

The result of the iterative optimization depends naturally on the number of iterations performed. While, for well-behaved optimization problems, a stopping criterion based on a predefined value of the metric can be used, this is not the case here. This is because it is not known a-priori which minimum value of the metric can be attained in practice. Therefore, Elastix only specifies a number of iterations as the stopping criterion, and leaves it up to the user to decide whether the solution is sufficiently converged (see Figure A5). Elastix uses the number of iterations as stopping criterion of the iterative process. The convergence therefore needs to be monitored by the user.

It is reasonable to expect that an increase in the number of iterations would be accompanied by an improvement in the recovered displacement field. However, Figure A5 shows that a plateau region is reached after 150 iterations. Further iterations do not appear to improve the result. It also shows that the correlation metric value as output by Elastix corresponds very well with our definition of the exact error (c.f, Equation (A1)). This indicates that the correlation metric value can be used to monitor convergence.
